# Clinical and Echocardiographic Characteristics of Neuroendocrine Tumours and Carcinoid Heart Disease: Insights from a Contemporary Canadian Cohort

**DOI:** 10.1016/j.cjco.2026.06.001

**Published:** 2026-06-15

**Authors:** Joshua Yoon, Cynthia Yeung, Jeffrey D. Wong, Janine Mazengarb, Christina L. Luong, Michael Y.C. Tsang, Teresa S.M. Tsang, Margot K. Davis, Kenneth Gin, John Jue, Parvathy Nair, Jonathan M. Loree, Darwin F. Yeung

**Affiliations:** a Department of Medicine, University of British Columbia, Vancouver, British Columbia, Canada; b Division of Medical Oncology, Department of Medicine, University of British Columbia, Vancouver, British Columbia, Canada; c Division of Cardiology, Department of Medicine, University of British Columbia, Vancouver, British Columbia, Canada

**Keywords:** neuroendocrine tumour, carcinoid heart disease, echocardiography, valvular heart disease

## Abstract

**Background:**

Neuroendocrine tumours (NETs) are uncommon cancers that secrete vasoactive hormones. Around half of those with metastatic small bowel NETs develop carcinoid syndrome, and historically, up to 50% develop carcinoid heart disease (CHD). We evaluated clinical characteristics, echocardiographic features, and outcomes of patients with NETs and CHD in a contemporary cohort to identify factors associated with CHD development and mortality.

**Methods:**

We identified patients with NETs who underwent an echocardiogram at a tertiary cancer centre–affiliated laboratory (2013-2023). Patients were classified based on whether they developed CHD. Clinical characteristics, echocardiographic data within 6 months of NET diagnosis, treatments, and outcomes were compared between CHD and non-CHD patients.

**Results:**

Over a 10-year period, 87 patients with NETs were identified (48% female; median age, 61 years), with 18 (21%) developing CHD. Primary small bowel NETs were more common with CHD (83% vs 55%, *P* = 0.032). Patients with CHD exclusively had right-sided valve involvement: 100% tricuspid, 61% pulmonary, and 0% mitral/aortic; 17% had a patent foramen ovale. Compared to non-CHD patients, CHD patients demonstrated higher right atrial volume index and right ventricular basal diameter, with more frequent right ventricular dysfunction. Patients with CHD had a lower median survival from the time of NET diagnosis (6.6 vs 13.4 years; *P* = 0.007). Among CHD patients, primary NET resection was associated with improved survival (*P* = 0.006).

**Conclusions:**

Within a contemporary cohort, approximately 1 in 5 patients with NETs develop CHD and exhibit only right-sided valve involvement. CHD was associated with increased mortality whereas primary NET resection was associated with improved survival.

## Résumé

**Contexte:**

Les tumeurs neuroendocrines (TNE) sont une forme peu fréquente de cancers qui sécrètent des hormones vasoactives. Environ la moitié des TNE métastatiques de l’intestin grêle entraînent un syndrome carcinoïde et, d’après les données historiques, jusqu’à 50 % entraînent une cardiopathie carcinoïde (CPC). Nous avons évalué les caractéristiques cliniques, les caractéristiques échocardiographiques et les résultats chez des patients présentant une TNE et une CPC dans une cohorte contemporaine, afin de déterminer les facteurs liés au développement de la CPC et à la mortalité qui lui est associée.

**Méthodologie:**

Nous avons recensé les patients présentant une TNE qui se sont prêtés à une échocardiographie dans un laboratoire tertiaire affilié à un centre de traitement du cancer (entre 2013 et 2023). Nous avons classé les patients selon qu’ils avaient ou non développé une CPC et comparé les caractéristiques cliniques, les données échocardiographiques dans les 6 mois suivant le diagnostic de TNE, les traitements et les résultats entre les patients atteints de CPC et les autres.

**Résultats:**

Sur une période de 10 ans, 87 patients présentant une TNE ont été recensés (48 % de femmes; âge médian : 61 ans), dont 18 (21 %) ayant développé une CPC. Les TNE primitives de l’intestin grêle étaient plus fréquentes en présence d’une CPC (83 % *vs* 55 %; *p* = 0,032). Les patients présentant une CPC présentaient exclusivement une atteinte valvulaire droite (valve tricuspide : 100 %, valve pulmonaire : 61 %, valve mitrale/aortique : 0 %, et 17 % présentaient un foramen ovale perméable). Comparativement aux patients qui n’étaient pas atteints d’une CPC, l’indice de volume de l’oreillette droite et le diamètre basal du ventricule droit étaient plus élevés chez les patients atteints d’une CPC, et la dysfonction ventriculaire droite était plus fréquente. La survie médiane à compter du diagnostic de TNE était plus faible chez les patients atteints d’une CPC (6,6 *vs* 13,4 ans; *p* = 0,007). Chez les patients atteints d’une CPC, la résection de la TNE primitive a été associée à une amélioration de la survie (*p* = 0,006).

**Conclusions:**

Dans une cohorte contemporaine, environ 1 patient présentant une TNE sur 5 développe une CPC et présente uniquement une atteinte valvulaire droite. La CPC a été associée à une mortalité accrue tandis que la résection de la TNE primitive a été associée à une amélioration de la survie.

Neuroendocrine tumours (NETs) are uncommon cancers. with an incidence of 8.5 cases per 100,000 persons.^1^ NETs originate predominantly in the gastrointestinal tract and lung, and they secrete vasoactive substances, such as serotonin and tachykinins, that can lead to carcinoid syndrome (CS).^2^ Characterized by flushing, bronchospasm, and diarrhea, CS has been estimated to occur in 20% of patients with NETs.^3^ Carcinoid heart disease (CHD) develops in 20%-50% of patients with CS, and it involves the development of thickened, retracted, and fixed valve leaflets resulting in significant valvular regurgitation and clinical heart failure.^2^^,^^4^ Right-sided heart valves (ie, tricuspid and pulmonary valves) are more often affected, as metabolism of vasoactive substances in the lungs reduces their exposure to left-sided valves.

The development of CHD historically has been associated with poor long-term prognosis, with one early study reporting a survival rate of no more than 31% over 3 years, and a median survival of 1.6 years, compared to 4.6 years in those without cardiac involvement.^5^^,^^6^ Liver metastases further increase the risk of CHD development, progression, and worse outcomes.^7^ Somatostatin analogs (SSAs) such as octreotide and lanreotide represent the first-line treatment for CS and unresectable midgut and pancreatic NETs, due to their anti-proliferative effects and ability to halt serotonin secretion.^8^^,^^9^ However, the impact of targeted therapies (eg, peptide receptor radionuclide therapy [PRRT]) on CHD progression and mortality remains unclear. In addition, other factors associated with improvement or worsening of valve function following initiation of treatment for CHD have yet to be elucidated fully.

Transthoracic echocardiography represents the cardiac imaging modality of choice for evaluation of CHD. As the development of CHD is associated with a lower survival rate in patients with advanced or unresectable NETs, earlier detection of CHD through routine surveillance echocardiography may improve outcomes by providing opportunities for more timely intervention. However, the current guideline-recommended frequency of surveillance echocardiography in patients with NETs without concomitant CHD has been variable, ranging from every 6 months to every 3 years.^10^, ^11^, ^12^ In addition, valvular surgery for patients with advanced CHD who meet current standard indications has been shown to improve survival, but the optimal timing for intervention remains unclear.^13^ A possible effect is that improved diagnostic capabilities, wider selection of medical therapies, and more sophisticated surgical techniques have changed the epidemiology, natural history, and clinical outcomes of patients with NETs and CHD.

In this study, we aimed to examine the clinical characteristics, echocardiographic features, and outcomes of patients with NETs, comparing those who did vs did not develop CHD, and examining the factors associated with CHD progression and adverse outcomes.

## Methods

### Study cohort

Patients with NETs who underwent transthoracic echocardiography at a tertiary cancer centre–affiliated echocardiography laboratory between 2013 and 2023 were identified. An advanced search of the echocardiography database was performed to identify reports that included the terms “carcinoid” or “neuroendocrine tumour” in either the indication field or within the findings and conclusions. The electronic medical records of these patients were reviewed, and patients were included in the study if they had a confirmed diagnosis of NET through tissue biopsy or surgical pathology.

### Clinical and echocardiographic characteristics

Baseline patient demographics and clinical characteristics were extracted from the electronic medical records. Demographic data included sex and age at the time of NET diagnosis. Comorbidities collected at the time of NET diagnosis included hypertension, dyslipidemia, diabetes mellitus, atrial fibrillation, coronary artery disease, heart failure, and preexisting valvular disease (eg, rheumatic heart disease, bicuspid aortic valve). Relevant biochemical parameters included the cardiac biomarkers—brain natriuretic peptide (BNP) and N-terminal prohormone of brain natriuretic peptide (NT-proBNP)—and the vasoactive peptides—chromogranin A (CgA) and 5-hydroxyindoleacetic acid (5-HIAA), with the first available result following NET diagnosis recorded. Normal levels of BNP and NT-proBNP were considered to be < 100 pg/mL and < 300 pg/mL, respectively.^14^

The baseline echocardiogram was defined as the first echocardiographic report within 6 months of NET diagnosis with an integrated assessment of 2-dimensional imaging, colour Doppler, and spectral Doppler data. Quantitative parameters of right-sided cardiac structure and function included right atrial volume index, right ventricular (RV) basal diameter, tricuspid annular plane systolic excursion (TAPSE), and RV systolic excursion velocity (S′). Visual assessment of RV systolic function was determined by 2 level III echocardiographers, with any decrease in function defined as abnormal. CHD was defined using a combined structural and functional approach consistent with established echocardiographic criteria, as previously described.^3^ In this framework, disease severity is determined by integrated assessment of valvular morphology, function, and RV involvement rather than isolated valve dysfunction alone, with the following semiquantitative scoring system (total score of 20): tricuspid valve morphology (0, normal; 1, thickened with reduced mobility; 2, thickened with severe immobility; 3, thickened and fixed); tricuspid regurgitation (TR; 0, none/trivial; 1, mild; 2, moderate; 3, severe including massive/torrential); pulmonic valve morphology (0, normal; 1, thickened with reduced mobility; 2, thickened with severe immobility; 3, thickened and fixed); pulmonic valve regurgitation (0, none/trivial; 1, mild; 2, moderate; 3, severe including massive/torrential); RV size (0, normal; 1, mild dilatation; 2, moderate dilatation; 3, severe dilatation); RV function (0, normal; 1, mild dysfunction; 2, moderate dysfunction; 3, severe impairment); as well as the presence (1) or absence (0) of diastolic forward flow in the pulmonary artery and systolic flow reversal in hepatic veins. Characteristic carcinoid valvular morphology was defined by the presence of leaflet thickening, retraction, and reduced mobility, often resulting in fixation of the valve leaflets in a semi-open position with impaired coaptation. Subvalvular involvement was also assessed when visible. These morphologic features predominantly involved the tricuspid and pulmonic valves, consistent with the known pathophysiology of serotonin-mediated endocardial fibrosis affecting right-sided cardiac structures. Echocardiograms were re-reviewed by 2 level III echocardiographers (D.Y., M.T.) who provided independent interpretations, including the following: (i) CHD diagnosis; (ii) RV size and systolic function; and (iii) tricuspid and pulmonary stenosis and regurgitation.

Data were collected on NET characteristics including primary tumour site and treatments administered, including surgical procedures (eg, primary resection, debulking surgery), medication therapies (eg, SSAs, PRRT, tyrosine kinase inhibitors (TKIs), chemotherapy), and radiation therapy.

### Clinical outcomes

Disease progression was quantitatively assessed based on estimated time from NET diagnosis to CHD diagnosis, NET diagnosis to death, CHD diagnosis to valvular replacement, and CHD diagnosis to death. Baseline echocardiograms of patients with NETs without clinical or echocardiographic evidence of CHD were compared to those of patients who were initially diagnosed with or eventually developed CHD. Time from NET diagnosis to death from any cause was compared between patients who did vs did not develop CHD. Among patients who developed CHD, characteristics were compared between those who survived < 2.5 years vs those who survived ≥ 2.5 years from CHD diagnosis or were alive by the end of study period; this threshold was selected as an exploratory cutoff approximating the median survival in our cohort and to facilitate balanced subgroup comparisons.

### Statistical analysis

Continuous variables were presented as median (interquartile range [IQR]) and were compared using the Mann–Whitney *U* test. Categorical variables were expressed as counts (%) and were compared using the χ^2^ test or Fisher’s exact test, as appropriate. Unadjusted Kaplan–Meier survival analysis was performed to compare CHD vs non-CHD groups, and differences were assessed using the log-rank test, with time zero set as the time of NET diagnosis, and the endpoint set as time of death or end of study period. Associations between treatments and survival were evaluated using univariate analyses, and statistical significance was defined as *P* < 0.05. Statistical analysis was performed using Prism software version 10.4.1 (GraphPad, San Diego, CA). The study was approved by the University of British Columbia Clinic Research Ethics Board.

## Results

### Patient characteristics

**Figure 1:**
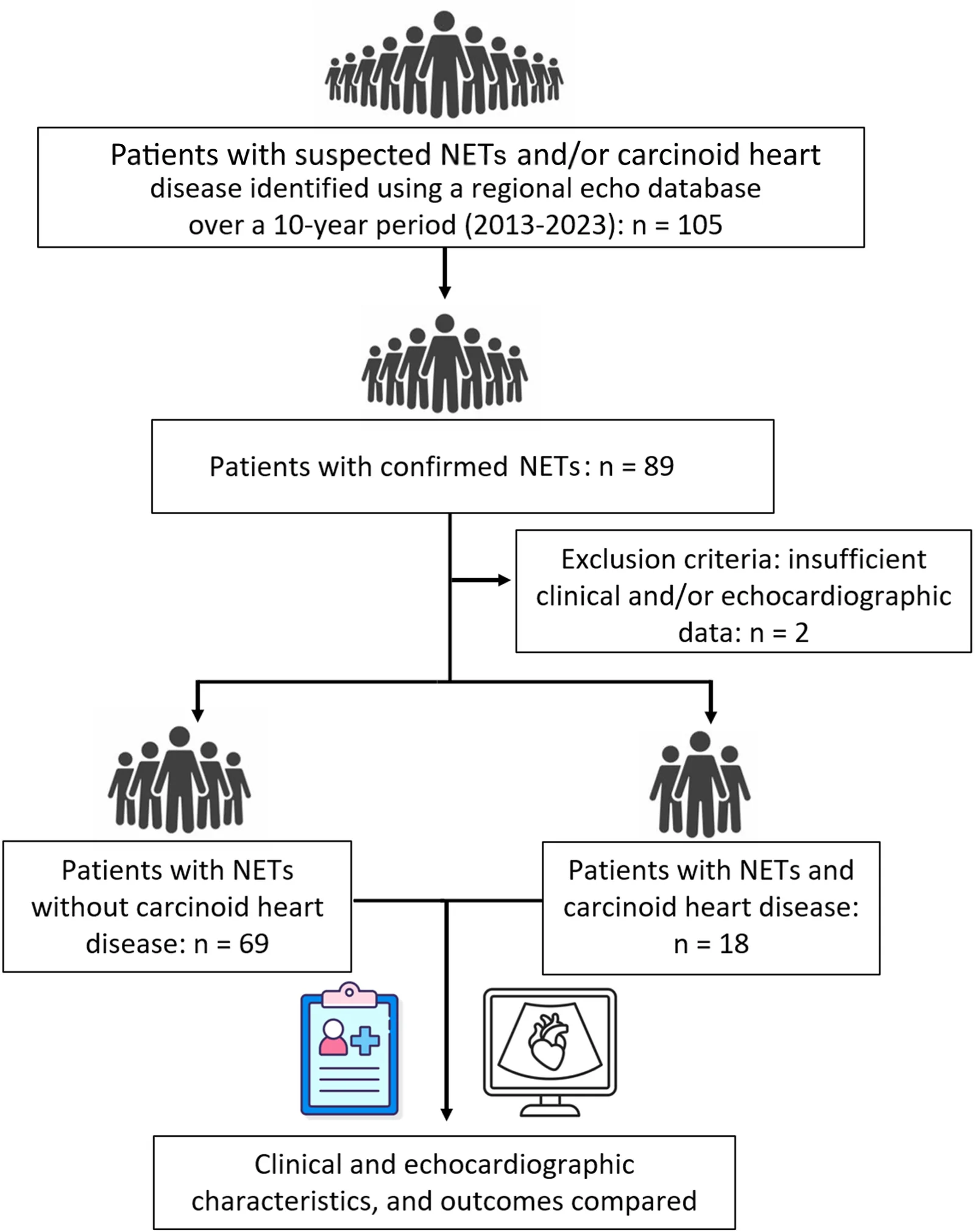
Flow chart of patients with neuroendocrine tumours (NETs) with vs without carcinoid heart disease.

**Figure 2:**
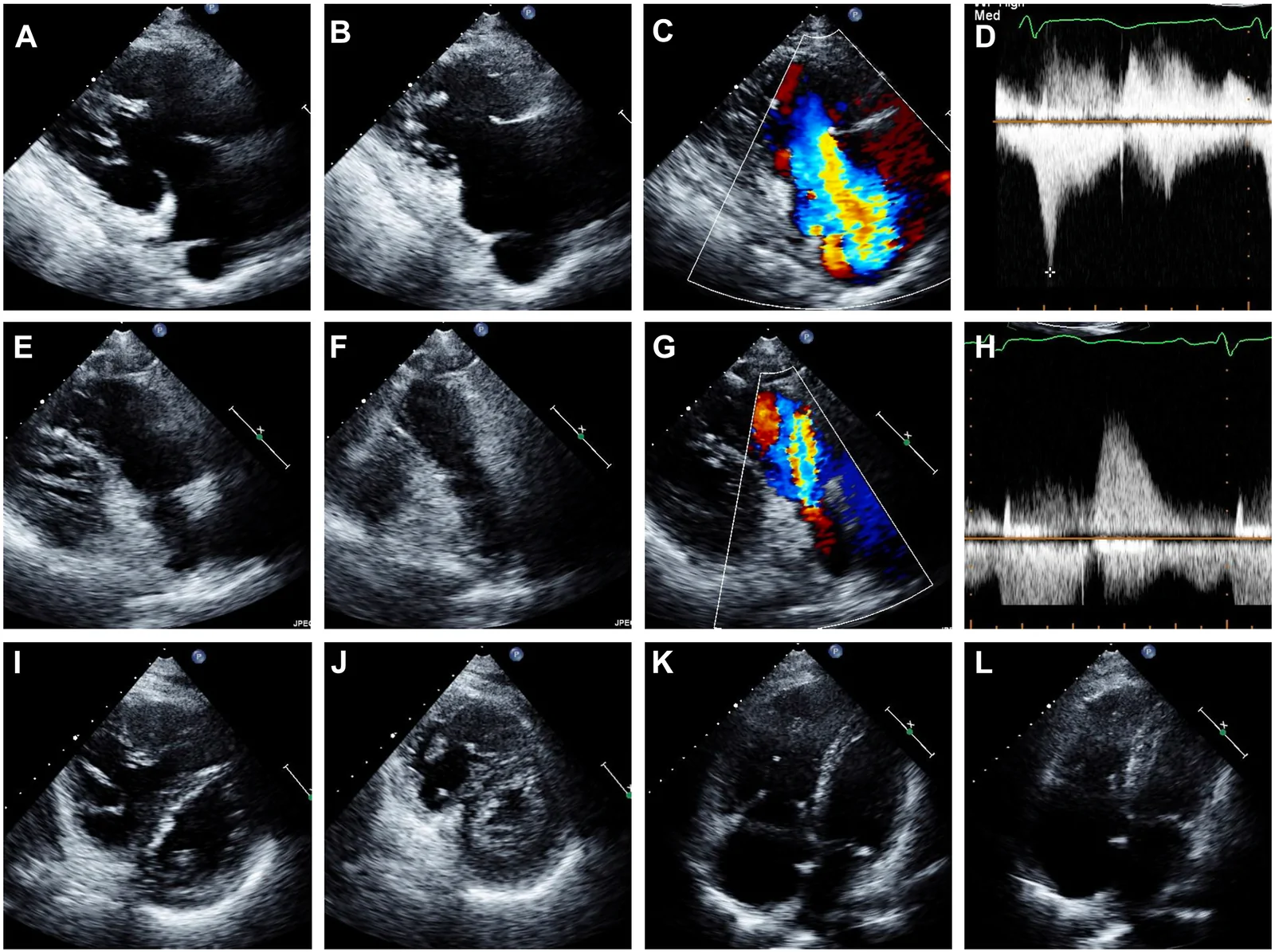
Echocardiographic findings of carcinoid heart disease. Representative transthoracic echocardiogram images of a patient in our cohort with carcinoid heart disease. Parasternal right ventricular (RV) inflow view shows fixed, retracted, and thickened tricuspid valve leaflets in (**A**) end-diastole and (**B**) end-systole. Evidence can be seen of severe tricuspid regurgitation based on (**C**) the large regurgitant jet area on colour Doppler and (**D**) the dense, triangular-shaped continuous wave Doppler signal in systole. Parasternal RV outflow view shows a similar appearance of the pulmonary valve in (**E**) end-diastole and (**F**) end-systole. Severe pulmonary regurgitation is suspected due to (**G**) the large regurgitant jet area on colour Doppler and (**H**) dense continuous wave Doppler signal with pressure half-time < 100 ms in diastole. Parasternal short-axis views show ventricular septal flattening in (**I**) end-diastole that is absent in (**J**) end-systole due to volume overload from severe right-sided valvular regurgitation. Similarly, apical 4-chamber views in (**K**) end-diastole and (**L**) end-systole show severe right atrial and RV enlargement from right-sided volume overload.

Figure 1 shows a flowchart of how patients with NETs were identified and included in the study cohort. Over a 10-year study period, 87 patients with NETs were identified, with 42 (48%) female patients and a median age of 61 years (IQR 53-69) at time of NET diagnosis. Within this cohort of patients with NETs, 31 (36%) developed or had CS at the time of evaluation, and 18 (58%) of these patients were either diagnosed with CHD at the time of NET diagnosis (8; 44%) or eventually developed CHD by the end of the study period (10; 56%) (median age 66 years [IQR 59-71]; 8 [44%] female). An example of CHD involvement in a patient with NET is shown in Figure 2. Among patients with CHD, 94% presented with metastatic NETs. The median time from NET diagnosis to development of CHD was 2.0 years (IQR 0-4.3).

### Clinical characteristics

The baseline clinical characteristics of the study cohort are shown in Table 1. Primary NETs of small bowel origin were more frequent in patients with CHD than they were in those who did not develop CHD (83% vs 55%, *P* = 0.032). At the time of NET diagnosis, hypertension was more common in patients with CHD than in those without CHD (55% vs 24%, *P* = 0.020). Of those who developed CHD, 17 (94%) had metastatic disease at the time of NET diagnosis. Metastatic disease was present in 64 patients (74%) and was predominantly liver-based (n = 43). Isolated lung metastases occurred in 3 patients, combined liver and lung metastases occurred in 3 patients, bone metastases occurred in 3 patients, and peritoneal metastases occurred in one patient. On cardiac review of systems, 5 patients (28%) were asymptomatic with no documented heart failure symptoms (ie, dyspnea, orthopnea, paroxysmal nocturnal dyspnea, peripheral edema) at the time of CHD diagnosis. No significant differences were present between the 2 groups in terms of cardiac medications prescribed, including angiotensin-converting enzyme inhibitors, angiotensin receptor blockers, mineralocorticoid receptor antagonists, or diuretics.

**Table 1:** Baseline characteristics of patients with neuroendocrine tumours

Characteristic	No CHD (n = 69)	CHD = 18)	*P*
Demographics			
Age, Y	60 [52, 69]	61 [53, 68]	0.807
Female	34 (48)	8 (44)	0.795
Cardiac comorbidities			
Hypertension	17 (24)	10 (55)	0.020
Dyslipidemia	10 (14)	1 (6)	0.447
Diabetes	11 (16)	2 (11)	0.999
Smoking	11 (16)	1 (6)	0.446
Coronary artery disease	6 (8)	1 (6)	0.999
Atrial fibrillation	3 (4)	3 (17)	0.100
Heart failure	0 (0)	6 (33)	< 0.001
NET characteristics			
Origin			
Small bowel	38 (55)	15 (83)	0.032
Large bowel	6 (9)	1 (5)	
Gastric	2 (3)	0 (0)	
Pancreatic	6 (9)	1 (5)	
Thymus	1 (1)	0 (0)	
Lung	6 (9)	0 (0)	
Endometrial/ovarian	1 (1)	1 (5)	
Unknown	9 (13)	0 (0)	
Metastatic disease			
Medications			
ACE inhibitors/ARBs	18 (26)	3 (17)	0.543
MRAs	1 (1.4)	0 (0)	
Loop diuretics	9 (13)	3 (17)	0.707
Echo characteristics			
LVEF, %	61	57	0.052
RAVI, mL/m^2^	24	41	0.001
RV systolic dysfunction	0 (0)	4 (22)	< 0.001
RV basal diameter, mm	34	40	0.006
TAPSE, mm	23.53	20.67	0.124
RV S′, cm/s	13.2	13.5	0.524

Data are expressed as number (percentage) for categorical variables and median (interquartile range) for non-normal continuous variables.

ACE, angiotensin-converting enzyme; ARB, angiotensin II receptor blocker; CHD, carcinoid heart disease; LVEF, left ventricular ejection fraction; MRA, mineralocorticoid receptor antagonist; NET, neuroendocrine tumour; RAVI, right atrial volume index; RV, right ventricular; TAPSE, tricuspid annular plane systolic excursion.

### Biochemical markers

**Figure 3:**
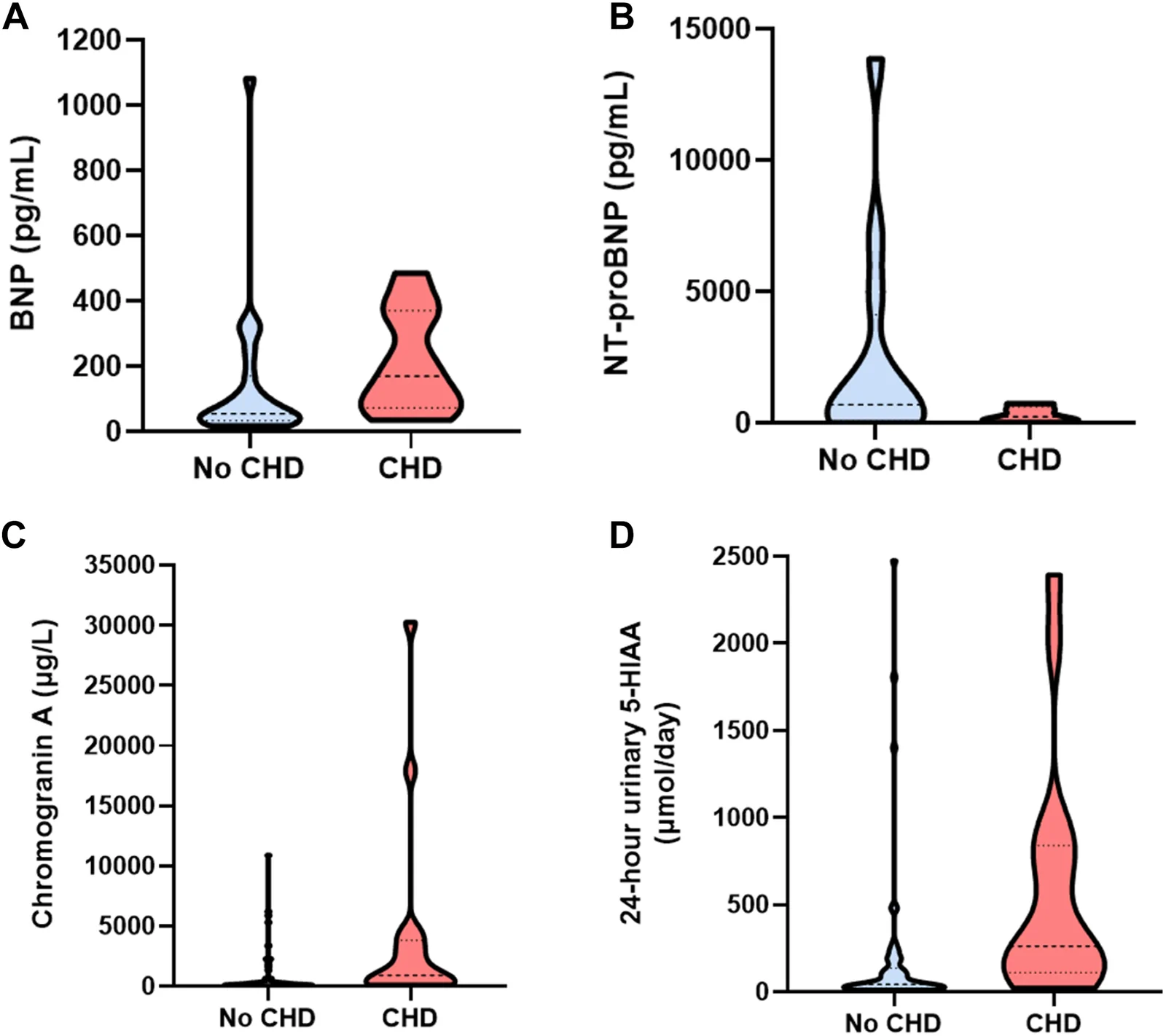
Violin plots of initial biomarker levels measured following neuroendocrine tumour diagnosis in patients with vs without carcinoid heart disease (CHD): (**A**) brain natriuretic peptide (BNP); (**B**) N-terminal prohormone of brain natriuretic peptide (NT-proBNP); (**C**) chromogranin A; and (**D**) 24-hour urinary 5- hydroxyindoleacetic acid (HIAA).

In patients with CHD, 13 (72%) had BNP or NT-proBNP levels measured, with a median time from NET diagnosis to measurement of 10.5 months (IQR 2.4-51.1). In patients without CHD, 35 (51%) had BNP or NT-proBNP levels measured with a median time from NET diagnosis to measurement of 42 months (IQR 13.2-120.8; (Fig. 3, A and B). At the time of first measurement of BNP or NT-proBNP following NET diagnosis, the CHD subgroup was more likely to have an abnormal value compared to the non-CHD subgroup (69% vs 34%; *P* = 0.030). Biomarker levels measured from the time of NET diagnosis, segregated by CHD status, are shown in Table 2. Patients with CHD had significantly higher levels of CgA (900 vs 152 μg/L, *P* = 0.002; Fig. 3C) and urinary 5-HIAA (262 vs 43 μmol/d, *P* < 0.001; Fig. 3D) than did those without CHD.

**Table 2:** Biomarkers in patients with neuroendocrine tumours with vs without carcinoid heart disease (CHD)

Biomarker∗	No CHD (n = 69)	CHD (n = 18)	*P*
Elevated BNP/NT-proBNP†	12 (34)	9 (69)	0.030
24-h urinary 5-HIAA (μmol/d)‡	43 [22, 125]	262 [154, 775]	< 0.001
Chromogranin A (μg/L)‡	152 [79, 366.25]	900 [174, 3206]	0.002

Data are expressed as number (percentage) for categorical variables and median [interquartile range] for non-normal continuous variables.

BNP, B-type natriuretic peptide; NT-proBNP, N-terminal prohormone of brain natriuretic peptide; 5-HIAA, 5-hydroxyindoleacetic acid.

∗ Reported biomarker values are the first measurements obtained following neuroendocrine tumour diagnosis.

† Normal reference ranges for BNP/NT-proBNP are as follows: BNP <100 pg/mL; NT-proBNP <300 pg/mL.

‡ Number of patients with 24-hour urinary 5-HIAA and chromogranin A levels: no CHD (n = 57); CHD (n = 17).

### Echocardiographic findings

Comparing patients with vs without CHD, the median time from NET diagnosis to their first echocardiogram was 8 months (IQR 0-30) and 5 months (IQR 2-33), respectively. The median carcinoid echocardiographic score for those who developed CHD compared to those who did not develop CHD was 10 (IQR, 6-14) and 0 (IQR, 0-1), respectively (*P* < 0.001). All patients with CHD exhibited right-sided valve disease only, with tricuspid valve involvement present in 100%, and pulmonary valve involvement present in 61%. No cases of significant mitral or aortic valve disease characteristic of CHD were seen within this cohort, including in the 3 patients (17%) who had a patent foramen ovale (PFO). At the time of baseline echocardiogram within 6 months from NET diagnosis, patients with CHD at the time of NET diagnosis or who eventually developed CHD within the study period demonstrated on average higher right atrial volume index (41 mL/m^2^ vs 24 mL/m^2^, *P* = 0.001), higher RV basal diameter (40 mm vs 34 mm, *P* = 0.006), and more frequent RV systolic dysfunction (22% vs 0%, *P* < 0.001). No significant differences occurred in quantitative measures of RV function (TAPSE or RV S′; Table 1).

### Survival analysis

**Figure 4:**
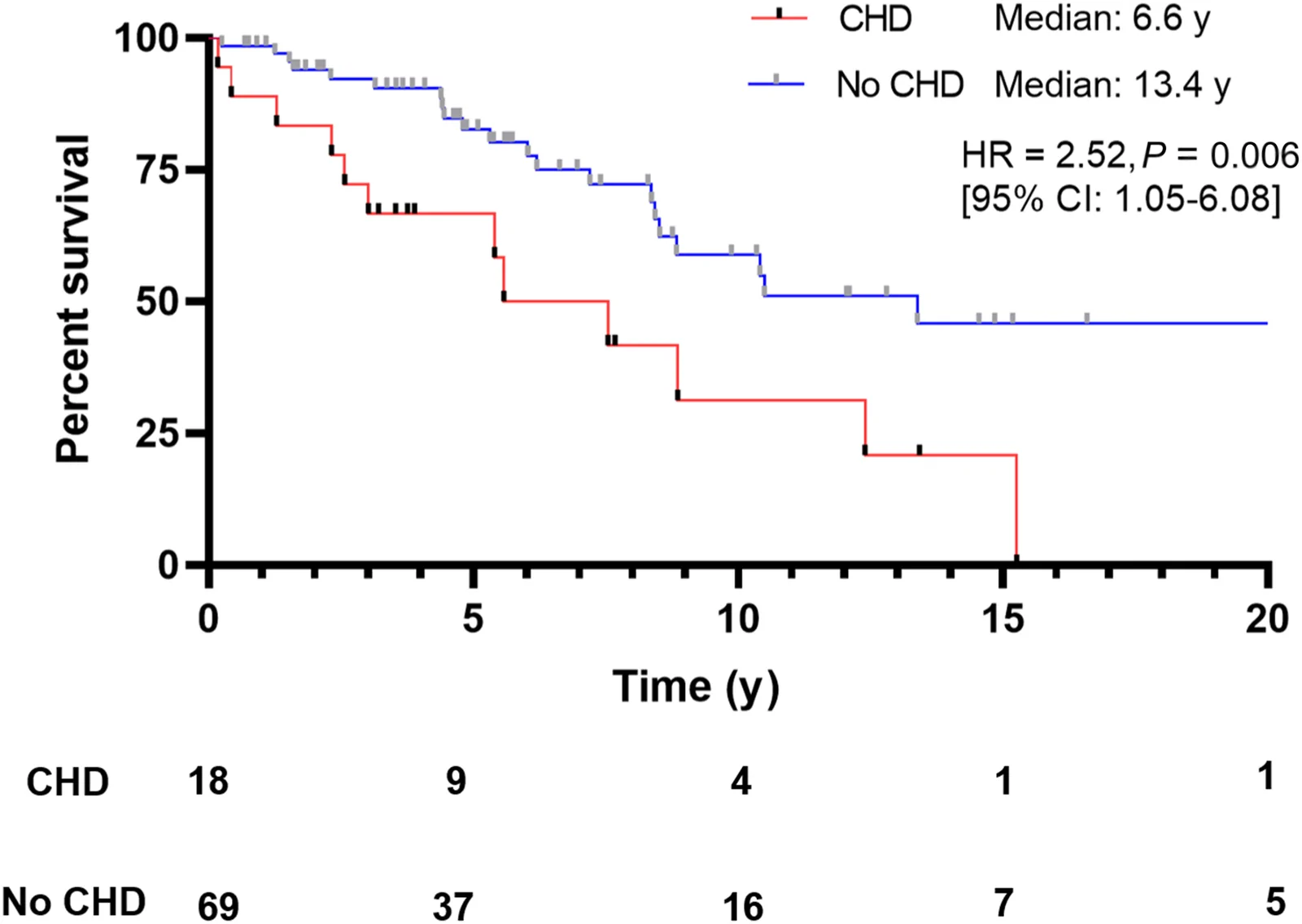
Kaplan-Meier survival curves for overall survival of patients with neuroendocrine tumours, segregated by development of carcinoid heart disease (CHD), from 2013-2023. CI, confidence interval; HR, hazard ratio.

The median survival time from NET diagnosis to death was significantly lower in patients with CHD compared to those without CHD (6.6 vs 13.4 years; *P* = 0.007; HR 2.52, 95% confidence interval [CI] 1.05-6.08; Fig. 4). NET progression was the primary cause of mortality in 75% of CHD patients, whereas 17% died due to refractory heart failure. The median survival from CHD diagnosis to death was 2.0 years (IQR 0.2-5.2). Among the subgroup of patients with CHD, a significantly lower rate of primary tumour resection occurred among those who survived < 2.5 years compared to those who survived ≥ 2.5 years following CHD diagnosis or were alive by the end of study period (*P* = 0.006).

### Therapeutic interventions and outcomes

Among the 18 patients with CHD, disease-modifying treatment strategies in order of frequency included SSAs (100%), primary tumour resections (72%), chemotherapy (39%), PRRT (33%), TKIs (28%), debulking surgery (17%), and radiation therapy (17%). Differences in treatment interventions between patients who survived < 2.5 years from NET diagnosis compared to those who survived ≥ 2.5 years from NET diagnosis or were alive by end of the study period are shown in Table 3. Tyrosine kinase inhibitors and radiation therapy showed trends toward improved survival, although statistical significance was not reached.

**Table 3:** Interventions in patients with carcinoid heart disease who survived < 2.5 years vs ≥ 2.5 years

Intervention	Survival < 2.5 Y (n = 8)	Survival ≥ 2.5 Y (n = 10)	*P*
Primary resection	3 (50)	10 (100)	0.006
Debulking surgery	1 (13)	2 (20)	0.999
Surgical valve replacement	3 (38)	5 (50)	0.664
Chemotherapy	3 (38)	4 (40)	0.999
Tyrosine kinase inhibitor	1 (13)	4 (40)	0.314
Radiation therapy	0 (0)	3 (30)	0.216
Peptide receptor radionuclide therapy	3 (38)	3 (30)	0.999

Data are expressed as number (percentage).

Surgical valve replacements occurred in 8 patients (44%) with CHD, with a median of 13.5 months (IQR 4-40) from CHD diagnosis to surgery. A combination of tricuspid and pulmonic valve replacements occurred in 6 patients, whereas 2 patients had isolated tricuspid valve replacement. Due to comorbidities and frailty, 6 patients (33%) with CHD who had an indication for valve replacement were deemed ineligible for cardiac surgery. No repeat valve interventions were documented during the study period. Of the 8 patients who underwent tricuspid and/or pulmonary valve replacement, survival outcomes were variable: 5 patients (63%) survived > 5 years after CHD diagnosis when treated with concomitant SSAs, PRRT, and/or chemotherapy; and 3 patients (38%) died within 2 years of CHD diagnosis due to right-sided heart failure with recurrent TR despite valve replacement.

Among the 10 patients with CHD who did not undergo surgery, 4 patients (40%) died within 1 year of CHD diagnosis, despite treatment with SSAs. One of these patients was recently started on lenvatinib (TKI) and chemotherapy but died from other causes. Another patient had worsening TR after PFO closure, with subsequent worsening RV function and death within 2 months. Of the remaining 6 patients with CHD who were alive during the last documented follow-up within the study period, their NET and valvular disease were wellcontrolled with a combination of SSAs, PRRT, TKIs, and/or radiation therapy; all of these patients had previously undergone primary tumour resection. Of note, 1 patient diagnosed with CHD 6 years after NET was alive at 3 years post-CHD diagnosis with disease well controlled on lenvatinib. Another patient had severe TR and PR but experienced significant improvement in valvular function and regurgitation after primary resection of their functional ovarian NET, remaining alive at 3.5 years post-CHD diagnosis.

## Discussion

In this contemporary cohort of patients with NETs, we observed several notable findings. Approximately 1 in 5 developed CHD over the 10-year study period, and notably all valvular lesions were right-sided. Patients who developed CHD had significantly worse survival compared to those without CHD, with progression of NET and resultant heart failure representing the leading causes of mortality in the CHD group. An important result is that among patients with CHD, those who underwent primary tumour resection and received multimodal systemic therapy had improved survival outcomes, compared to CHD patients who did not receive these interventions.

The epidemiologic patterns of CHD observed in our modern cohort may reflect a shift in disease landscape that may be driven by improved surveillance and advancements in NET-directed therapies. Historically, CS occurred in approximately 20% of patients with NETs, and CHD occurred in up to half of patients with CS, with left-sided involvement seen in up to 10% of cases, often in the presence of a right-to-left shunt.^3^^,^^15^, ^16^, ^17^, ^18^, ^19^, ^20^ In our cohort, 20% of patients with NETs developed CHD, and cardiac involvement was limited to right-sided valves with no left-sided disease. This result may be due to earlier disease detection and improved medical management in the contemporary era, particularly with the widespread use of therapies that reduce tumour burden, which potentially mitigate the severity and extent of cardiac involvement. However, comparison of CHD prevalence across studies should be done cautiously due to substantial heterogeneity in cohort composition, referral patterns, screening strategies, and the proportion of patients with metastatic disease or carcinoid syndrome.

The prognosis of patients with NETs who did go on to develop CHD continues to be poor, even in this contemporary cohort. Consistent with prior studies, CHD was associated with a higher risk of death compared to the risk among patients who did not develop CHD, predominantly due to NET progression and heart failure.^15^^,^^16^^,^^18^ An important finding is that more than a quarter of patients who developed CHD in our cohort were asymptomatic at the time their valvular disease was identified, highlighting the importance of routine surveillance in patients with NETs. Indeed, prior studies have described similar patients who exhibit minimal symptoms despite having moderate-to-severe TR.^17^^,^^21^^,^^22^ These findings highlight the importance of screening strategies to detect CHD earlier.

Routine biochemical testing of cardiac biomarkers or vasoactive peptides may offer a convenient way to screen for CHD development or monitor disease progression.^5^^,^^23^ NT-proBNP level has been shown to have both diagnostic and prognostic significance for cardiac involvement in patients with NETs, with median levels being significantly higher in those with CHD than in those without CHD.^24^^,^^25^ In our cohort, patients who developed CHD were more likely to have had an elevated BNP or NT-proBNP level at the time of initial NET diagnosis, as well as higher levels of serotonin biomarkers (eg, urinary 5-HIAA) and neuroendocrine markers (eg, CgA).^11^^,^^26^^,^^27^ These findings have important surveillance implications for patients with NETs, as significant biomarker elevation could alert clinicians to perform baseline or more frequent repeat cardiac imaging to screen for CHD development or progression.

Current guidelines recommend echocardiographic surveillance every year in patients with significant (> 5 times the upper limit of normal) elevation in serotonin or 5-HIAA levels, and every 6-12 months in patients with CS.^10^^,^^11^ More frequent imaging surveillance to monitor progression of valvular dysfunction, as well as RV remodelling and dysfunction, may be reserved for patients with established CHD. Nevertheless, no consensus among experts has been reached regarding the optimal frequency of echocardiographic follow-up, and this therefore represents an area for further investigation.

On baseline or follow-up echocardiogram of patients with CHD who have significant right-sided valve disease, accurate assessment of RV function is of utmost importance to guide management and inform prognosis, but it can be challenging. For instance, in the current study, the average TAPSE and RV S′ in patients with vs without CHD were found to be similar and within normal limits. These longitudinal RV indices are load-dependent and may overestimate RV function in the context of severe TR, which may result in altered hemodynamics, right heart maladaptation, and distortion of the tricuspid annulus.^28^ In contrast, patients with CHD in our cohort were on average found to have worse RV function than those without CHD, based on expert visual assessment of RV function, which takes into account RV free wall motion. Quantitative measures of RV function that incorporate RV free wall motion, such as fractional area change or RV free wall longitudinal strain, could potentially overcome the limitations of TAPSE and RV S′. However, these additional parameters have their own challenges, as they can be limited by suboptimal image quality and subject to significant interobserver variability, which precluded their use in the current retrospective study. Assessment of RV function therefore must not rely on a single variable but should integrate both visual estimate and any quantitative parameters that are available to be measured reliably in a given study.

Screening for CHD and monitoring for disease progression may have even more value in the contemporary era, as more medical treatment options are now available for patients with NETs, including SSAs, PRRT, and targeted chemotherapy agents. Our findings reinforce the notion that comprehensive systemic therapy aimed to lower tumour burden could potentially prevent CHD development, or at least delay its progression.^29^ Indeed, several patients in our study were able to defer valve surgery for a period of time, as their CS and hormonal burden were effectively controlled with medical therapies. In addition, a trend toward improved survival occurred in patients receiving multimodal NET treatments, compared to those who underwent valve surgery alone. Aggressive treatment of the underlying NET, either through medications or surgical resection of the primary tumour, may therefore modify the course of CHD in some cases, presumably by reducing the burden of vasoactive peptides.^30^ Prior studies have shown that patients with delayed NET treatment have higher rates of CHD and increased mortality.^29^ Further prospective studies are needed to clarify the impact of specific systemic treatments on CHD outcomes.

Therapies that specifically aim to lower tumour burden may play a significant role in CHD management. In our cohort, primary tumour resection was significantly associated with improved survival among patients with CHD. In particular, one patient with severe TR demonstrated significant improvement in valvular function after resection of the ovarian NET and initiation of SSA therapy.^30^ Removing the NET or reducing its mass at an earlier stage may limit the ongoing release of vasoactive substances that drive cardiac damage and has been observed in other case reports.^31^^,^^32^ In addition, patients with CHD who have undergone hepatic resection are known to have decreased risk of valvular disease progression and longer survival.^33^ Patients in our study who had tumour debulking similarly showed a trend toward improved survival, although this could represent selection bias, as patients selected for surgery may inherently have less aggressive disease or better performance status. Nevertheless, findings from our study and prior studies suggest that resection of the primary tumour and tumour debulking could improve oncologic and cardiovascular outcomes.

Although in rare instances, valve function has improved following primary tumour resection, valvular dysfunction is more often progressive in patients with CHD. When valve disease becomes severe, guidelines recommend valve replacements in patients with symptoms and an expected survival greater than 1 year.^34^ Improved survival in the 1990s has been credited largely to the wider use of surgical valve replacement, which has been shown to be independently associated with a reduced risk of death in patients with CHD.^6^ Despite these advances, a more recent meta-analysis showed that pooled mortality after valve surgery remained as high as 31% at 1 year and 56% at 2 years postintervention.^35^, ^36^, ^37^ Despite the small size of our cohort, our findings were similar, with mortality at 2 years post-surgery falling within the range of prior estimates at 38%. Of note, a third of patients with CHD in our cohort had an indication for valve replacement but were deemed ineligible for cardiac surgery, as their perioperative surgical risk was considered prohibitive. Successful transcatheter tricuspid and pulmonary valve interventions in this population have been reported previously, but have not yet been systematically studied.^38^, ^39^, ^40^ Whether transcatheter intervention could be performed routinely in these high-risk patients as an alternative to surgery warrants further study.

### Limitations

Our study provides informative insights, but it has important limitations that need to be considered when interpreting our findings. First, as a retrospective single-centre study, this work is subject to the usual limitations of selection bias, missing data, and unmeasured confounders. Second, patient identification was based on an echocardiography database, which may have introduced selection bias. Patients with NETs who did not undergo echocardiographic evaluation would not have been captured, and some patients may have been missed if the indication for imaging did not include relevant keywords. In contrast, our cohort may have been enriched with patients at increased risk of CHD, particularly those with CS and/or metastatic disease who may have been preferentially referred for echocardiographic screening. Accordingly, this study cannot be considered to represent a population-based NET cohort, but rather includes a group of patients with NETs who underwent evaluation at a tertiary care echocardiography laboratory affiliated with our provincial cancer care centre; therefore, prevalence estimates observed in this study should be interpreted within this context. Third, given the relative rarity of CHD, the sample size of our cohort was modest, which may have limited the statistical power and the ability to detect smaller effect sizes. Fourth, differences in NET characteristics and stage of disease likely influenced the timing and selection of treatment strategies, limiting our ability to isolate the independent contribution of specific therapies to CHD development and survival outcomes. An arbitrary cutoff of 2.5 years, based on estimated median survival, was used to compare characteristics of patients with CHD who survived less than or longer than this duration. Fifth, most patients in this cohort did not have routine evaluation of RV fractional area change or RV free wall longitudinal strain, especially those with suboptimal image quality and those with CHD diagnosed earlier in the study period. Finally, screening of PFO with bubble study was not systematically performed but was completed only when requested or suspected based on standard echocardiographic imaging. Although the number of PFOs may therefore have been underestimated, this is unlikely to have been clinically relevant given the lack of left-sided disease seen in our cohort.

## Conclusions

Our study provides a contemporary perspective on CHD patterns and prognosis in patients with NETs. Improved diagnostic techniques and enhanced therapeutic options may have resulted in the lack of left-sided valve involvement in our cohort. However, overall survival remains lower in patients with CHD, compared to those without, predominantly due to NET progression and heart failure. Surgical valve replacement had the potential to improve survival, especially when combined with adjunct disease-modifying therapies, but it was not available to high-risk patients and still carried a relatively high 2-year post-surgical mortality rate, similar to rates reported in recent meta-analyses. Further studies are needed to determine the optimal screening, surveillance, and treatment strategies in these patients with CHD.

## Ethics Statement

The study was approved by the University of British Columbia Clinic Research Ethics Board.

This research study adhered to the Tri-Council Policy Statement: Ethical Conduct for Research Involving Humans – TCPS 2 (2022).

## Patient Consent

The authors confirm that patient consent is not applicable to this article as it was a retrospective study using de-identified data. Consent was not required from each patient.

## Funding Sources

The authors have no funding sources to declare.

## Disclosures

J.M. was supported by a grant from the Heart Foundation of New Zealand. C.L.L. received salary support from the Vancouver Coastal Health Research Institute and the Heart & Stroke Foundation. D.F.Y. received salary support from the Vancouver Coastal Health Research Institute. The other authors have no conflicts of interest to disclose.
